# Seasonal variation in multiple sclerosis relapse

**DOI:** 10.1007/s00415-017-8485-0

**Published:** 2017-04-19

**Authors:** Katharine Harding, Kate Tilling, Claire MacIver, Mark Willis, Fady Joseph, Gillian Ingram, Claire Hirst, Mark Wardle, Trevor Pickersgill, Yoav Ben-Shlomo, Neil Robertson

**Affiliations:** 1 Institute of Psychological Medicine and Clinical Neuroscience, Cardiff University, University Hospital of Wales, Heath Park, Cardiff, CF14 4XW UK; 2 Department of Neurology, Helen Durham Centre for Neuroinflammatory Disease, University Hospital of Wales, Heath Park, Cardiff, CF14 4XW UK; 3 Department of Neurology, Morriston Hospital, Heol Maes Eglwys, Morriston, Swansea, SA6 6NL UK; 4 School of Social and Community Medicine, Canynge Hall, 39 Whatley Road, Bristol, BS8 2PS UK; 5 Department of Neurology, Royal Gwent Hospital, Cardiff Road, Newport, NP20 2UB UK

**Keywords:** Multiple sclerosis, Epidemiology, Relapses, Seasonal

## Abstract

Relapses are a characteristic clinical feature of multiple sclerosis (MS), but an appreciation of factors that cause them remains elusive. In this study, we have examined seasonal variation of relapse in a large population-based MS cohort and correlated observed patterns with age, sex, disease course, and climatic factors. Relapse data were recorded prospectively in 2076 patients between 2005 and 2014. 3902 events were recorded in 1158 patients (range 0–24). There was significant seasonal variation in relapse rates (*p* < 0.0001) and this was associated with monthly hours of sunshine (odds ratio OR 1.08, *p* = 0.02). Relapse rates were highest in patients under the age of 30 (OR 1.42, *p* = 0.0005) and decreased with age. There was no evidence of different relapse rates for males compared to females (OR 0.90, *p* = 0.19). Identification of potentially modifiable environmental factors associated with temporal variation in relapse rates may allow alteration of risk on a population basis and alteration of outcome of established disease once established. Future epidemiological studies should examine dynamic environmental factors with serial prospective measurements and biological sampling. Significant seasonal differences in relapse rates highlight the importance of environmental factors in disease expression and should be taken into account when planning clinical trials in which relapse frequency is an outcome. In addition, identification of potentially modifiable factors associated with this variation may offer unique opportunities for alteration of risk of relapse and long-term outcome on a population level, and suggest putative biological mechanisms for relapse initiation.

## Introduction

Relapses are a key feature of multiple sclerosis (MS); most patients will experience at least one relapse over the course of their disease, and the majority of currently available disease modifying therapies (DMTs) are targeted at reducing their frequency. A relapse is the clinical manifestation of acute inflammation and focal demyelination in a clinically eloquent region of the central nervous system (CNS), leading to a discrete episode of neurological dysfunction which may recover fully or partially [4]. However, identification of triggers that instigate the inflammatory cascade, and subsequent demyelination and gliosis, remains elusive.

A seasonal variation in relapses has been demonstrated in some [2, 7, 9, 23, 24, 26, 29, 31] but not all [8, 17, 18, 22] previous studies. However, many of these are powered to detect only the largest deflections from a random relapse distribution, whilst in larger pooled studies, up to 38% of relapses have been excluded as the date of relapse was unknown, leading to difficulties in interpretation of results [26]. Where seasonal variation has been identified, peak frequencies in spring and summer months irrespective of latitude have generally been observed [2, 7, 9, 17, 23], and this pattern has also been supported by magnetic resonance imaging data [16]. Direct comparison or meta-analysis of these studies, which would allow detailed interrogation of available data and population sub-groups, is prevented by variation in methodology and geographical location. Clarification of seasonal relapse patterns would provide direction for future research into environmental factors relevant to MS and may suggest putative mechanisms for relapse initiation.

To date, there have been no large-scale studies of seasonal relapse variation in northern Europe, an area which has the highest prevalence of MS in the world. In this study, we have collected relapse data on a large population-based cohort of patients within south east Wales over 10 years to examine seasonal variation in relapse frequency, and have compared observed patterns with selected climatic variables.

## Methods

### Patient recruitment

The neuroinflammatory unit at the University Hospital of Wales serves the population of Cardiff and Vale and Cwm Taf Health Boards, comprising the city of Cardiff and the surrounding rural and ex-mining areas of the Vale of Glamorgan and the Rhondda valley, while the city of Newport and its surroundings including Monmouthshire are served by clinics based at the Royal Gwent Hospital. A cross-sectional epidemiological study establishing the region as a high prevalence area for MS was first performed in 1985 [28], and has periodically been updated over subsequent years [10]. Since 1999, prospective longitudinal clinical data have been collected on all patients referred to the regional neuroinflammatory services. At first presentation, information is collected on demographics, details of onset of disease, current Expanded Disability Status Scale (EDSS) [13] score, current disease course, and relapses. Patients are reviewed routinely every 6–12 months, when further data are collected on current disease course, EDSS score, medication and its effects, and whether the patient has had a relapse since the last review. In addition, there is a weekly rapid access clinic which patients can directly access for assessment and treatment of new neurological symptoms including relapses. Relapses are defined as acute or subacute deteriorations in neurological function in the absence of fever or infection, with partial or full recovery [15].

**Table 1:** Patient characteristics

	All patients	Patients with events	Patients without events
Total number	2076	1158	918
Female (%)	1450 (69.8)	851 (73.5)	599 (65.3)
Current disease course:			
RRMS (%)	864 (41.6)	711 (61.4)	153 (16.7)
SPMS (%)	926 (44.6)	355 (30.7)	571 (62.2)
PPMS (%)	238 (11.5)	92 (7.9)	146 (15.9)
Mean study follow-up (SD)	7.2 years (4.7)	6.8 years (4.5)	7.8 years (4.9)
Mean age at onset (SD)	32.8 years (11.0)	32.6 years (10.8)	33.0 years (11.2)
Mean current age (SD)	54.2 years (13.8)	47.5 years (11.6)	62.6 years (11.6)
Mean disease duration (SD)	18.8 years (12.0)	13.5 years (9.1)	26.6 years (11.6)

*RRMS* relapsing–remitting MS, *SPMS* secondary progressive MS, *PPMS* primary progressive MS, *SD* standard deviation

All patients provided written informed consent. The study was approved by the South East Wales Research Ethics Committee (ref 05/WSE03/111). All patients were included in this study, unless the date of their most recent review was before 1st January 2005, or if onset of disease was after 31st December 2014.

### Climate data

Average data for south east Wales on minimum temperature, maximum temperature, hours of sunshine, and millimeters of rainfall for each month between January 2005 and December 2014 were downloaded from the Met Office website (http://www.metoffice.gov.uk/public/weather/climate-historic/tab=climateHistoric, accessed 29th February 2016). UV index data for the UK between January 2005 and December 2014 were downloaded from the Department for Environment, Food and Rural Affairs (DEFRA) (http://ozone-uv.defra.gov.uk/uv/data_search.php, accessed 29th February 2016).

### Statistical analysis

Patients contributed to each month of the study between onset of first MS symptoms and the most recent date of review by neuroinflammatory services. Where onset was before 1st of January 2005, patients contributed from that date, but if onset was after 1st January 2005, they contributed from the date of onset of symptoms. If patients were reviewed after 31st December 2014, they contributed to all months up to this date, but if the date of last encounter was earlier than 31st December 2014, data were censored at the time of the last encounter. For example, a patient with onset of disease in February 2006 who is still under review and was last seen in August 2016 would contribute to all months of the study between February 2006 and December 2014. A patient with onset in March 1990 who died in November 2010 would contribute to all months of the study between January 2005 and November 2010.

Initially, relapse counts were analysed by month using a Poisson regression model. The median number of relapses per month was used as the reference. Following this, logistic regression, clustered by patient, was used to analyse contribution of climate variables (hours of sunshine, rainfall, minimum and maximum monthly temperature, and UV index) to odds of relapse in any given month. Any relapse where the date was unknown was excluded from analysis. Current age, sex, and current disease course (relapsing–remitting or progressive) were included as co-variates. Our data set consists of observations for each month, for all patients who were present in the study during that month. A logistic regression model is used, because for each patient and for each month, they have a “1” if they had a relapse in that month, and a “0” otherwise. In view of previous studies suggesting a lagged effect of UV index [29], models were generated using raw data and also 1 and 2 month lags for climate variables. The Akaike Information Criterion (AIC) was used to select the best-fitting model.

Association between climate and recovery from relapse was also tested using clustered logistic regression. Relapses were classified according to whether the patient returned to the same level of disability recorded prior to relapse, or not. Only patients with relapsing disease were included in this model, to avoid the potential confounding effect of background progressive disease on recovery from relapses.

To analyse whether season of onset was associated with longer term outcomes, onset events were grouped into seasons: December, January, and February classified as winter; March, April, and May as spring; June, July, and August as summer; and September, October, and November as autumn. Kaplan–Meier survival analysis and Cox proportional hazards regression were used to analyse the relationship between season of onset of MS and time to EDSS 3.0 and 4.0.

R v3.2.2 was used for all analyses [20].

## Results

### Patients

2076 patients were included in this study, of whom 1450 (69.4%) were female. On the 1st of January 2005, 374 patients were aged less than 30, 528 were between 30 and 39, 534 were between 40 and 49, and 640 were 50 or older. Disease course at onset was relapsing in 1790 patients (86%) and progressive in 238 (11.5%). Of relapsing onset patients, 926 (52%) subsequently converted to SPMS. Mean duration of patient follow-up in the study was 7.8 years.

1158 patients (55.8%) experienced one or more relapses; the remaining 918 (44.2%) had no relapses during the study. 301 patients had one, 246 had two, 174 had three, and 437 had more than three relapses. Median number of relapses was 1 (interquartile range 0–3). Mean annualised relapse rate (ARR) for the cohort was 0.34 and 0.61 for those who had at least one relapse during the study period (Table 1).

### Relapses

Over the 10 year study period, there were 3902 events in 1158 patients. 528 (13.5%) were first events, and the remainder were subsequent relapses. The date for 234 (6.0%) of the events was unknown and could not be determined even after review of the medical notes. These events were not included in the statistical analysis. 937 relapses (24.0%) occurred in male patients, and 1292 (33.1%) in patients with progressive disease.

**Fig. 1:**
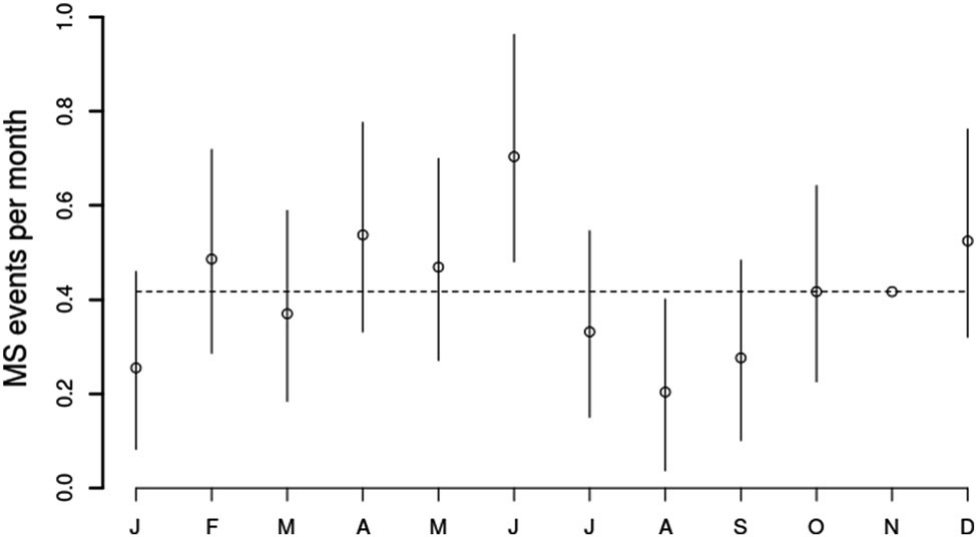
*Line graph* showing rate ratios and confidence intervals for MS events in each month, as calculated using Poisson regression

There was a sinusoidal pattern of number of events per month, with a peak in June (373 events, rate ratio 1.22, 95% confidence interval CI 1.05–1.42, *p* = 0.01), and a trough in August (256 events, rate ratio 0.84, 95% confidence interval 0.71–0.99, *p* = 0.04) (Fig. 1). The median number of events in any calendar month was 305. A summary of relapse numbers by month and corresponding climate variables for south east Wales are provided in Table 2.

**Table 2:** Monthly summary of relapses and climate variables, 2005–2014

Month	Relapses	Patient-months observed	Onset events	Mean max temp (°C)	Mean min temp (°C)	Mean rainfall (mm)	Mean hours of sunshine	Mean UV index
Jan	271	8645	32	8.3	2.4	102.4	57.0	0.23
Feb	321	10182	42	8.5	2.3	63.4	71.8	0.39
Mar	295	7323	35	11.1	3.2	62.6	126.9	0.77
Apr	334	7941	48	14.8	5.5	53.4	171.6	1.25
May	318	8156	45	17.3	8.3	80.2	196.9	1.6
Jun	373	9662	41	20.3	11.1	71.5	204.6	1.78
Jul	286	7184	37	22.2	13.0	87.6	217.1	1.85
Aug	256	6274	29	20.9	12.4	84.5	172.9	1.54
Sep	273	6736	31	19.4	10.8	66.5	138.3	1.24
Oct	304	5778	35	15.7	8.6	103.3	100.6	0.65
Nov	306	5628	37	11.2	4.6	107.6	67.8	0.31
Dec	331	8464	32	8.3	1.8	101.6	49.9	0.19

### Clinical variables and relapses

**Fig. 2:**
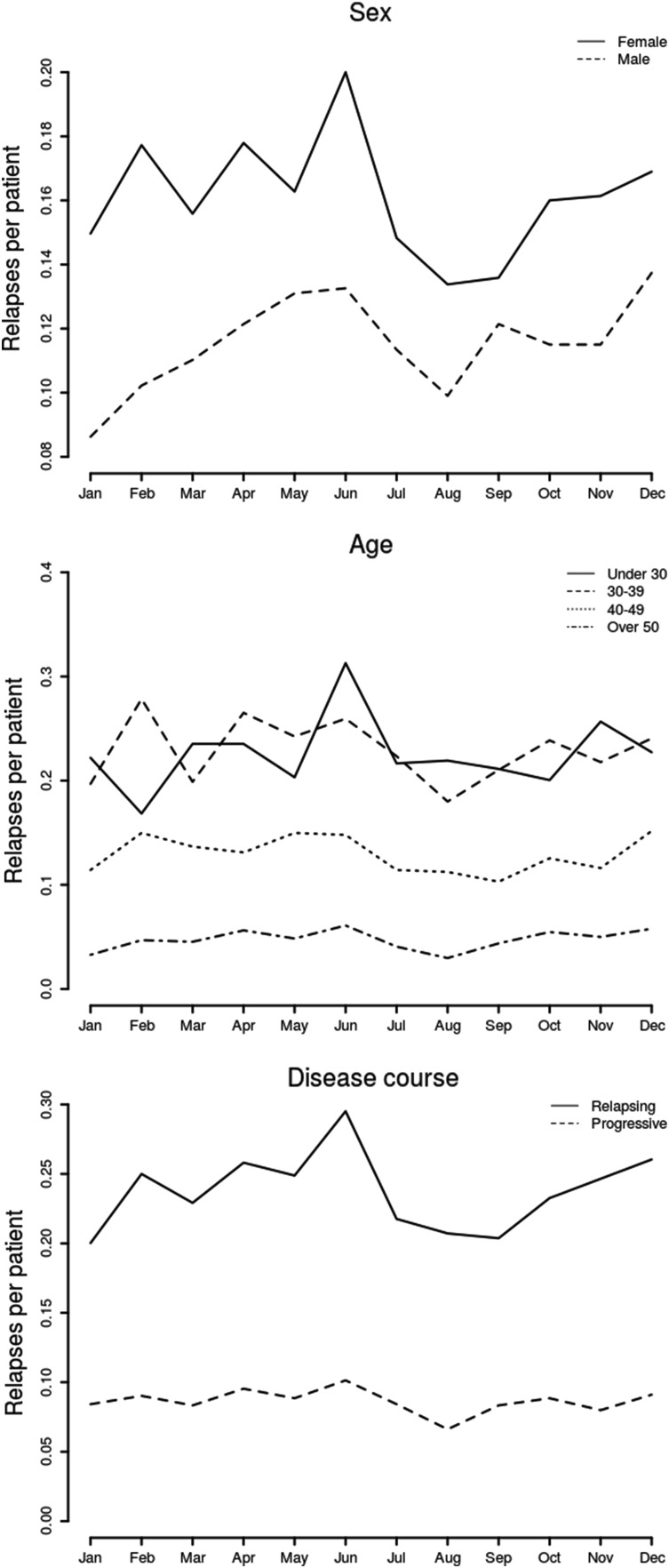
*Line graph* showing relapses per month, grouped by sex (*top*), age at relapse (*middle*), and disease course (*bottom*)

A relapse was more likely to occur in relapsing than progressive MS (odds ratio [OR] 1.32, 95% CI 1.12–1.54, *p* = 0.0005). MS event frequency decreased with age (30–39: reference; under 30: OR 1.42, 95% CI 1.16–1.73; 40–49: OR 0.64, 95% CI 0.53–0.76; 50 and over: OR 0.27, 95% CI 0.22–0.33, *p* < 0.0001). There was no difference in pattern of relapses between men and women (male: OR 0.90, 95% CI 0.77–1.05, *p* = 0.19), and a similar age-related seasonal pattern was seen in both sexes (Fig. 2). There was no interaction between age group and sex (*p* = 0.61).

### Climate variables and seasonal relapse pattern

The seasonal variation in relapses was confirmed in the logistic regression model (*p* < 0.001). The best-fitting model included hours of sunshine (OR 1.08, 95% CI 1.01–1.16, *p* = 0.023), age group (under 30: OR 1.42, 95% CI 1.16–1.73; 30–39: reference; 40–49: OR 0.64, 95% CI 0.53–0.76; 50 or older: OR 0.27, 95% CI 0.22–0.33, *p* < 0.0001), and disease course (progressive disease: OR 0.76, 95% CI 0.65–0.89, *p* = 0.00059) (Table 3). There was no association between relapses and sex (OR 0.90, 95% CI 0.77–1.05, *p* = 0.19). Models including other climate variables (rainfall, maximum or minimum temperature, or UV levels) had a less good fit as measured by AIC than the model including sunshine. The final model including hours of sunshine did not account for all the variance in relapse pattern observed.

**Table 3:** Final model for seasonal and clinical factors associated with relapse in patients with MS

Variable	Odds ratio (95% confidence interval)	*p* value
Sunshine	1.08 (1.01–1.16)	0.023
Age group		
<30 years	1.42 (1.16–1.73)	<0.0001
30–40 years	Reference	
40–50 years	0.64 (0.53–0.76)	
Over 50 years	0.27 (0.22–0.33)	
Sex (male)	0.90 (0.76–1.06)	0.19
Disease course		
RRMS	Reference	0.00059
Progressive	0.76 (0.65–0.89)	

### Climate variables and recovery from relapse

**Fig. 3:**
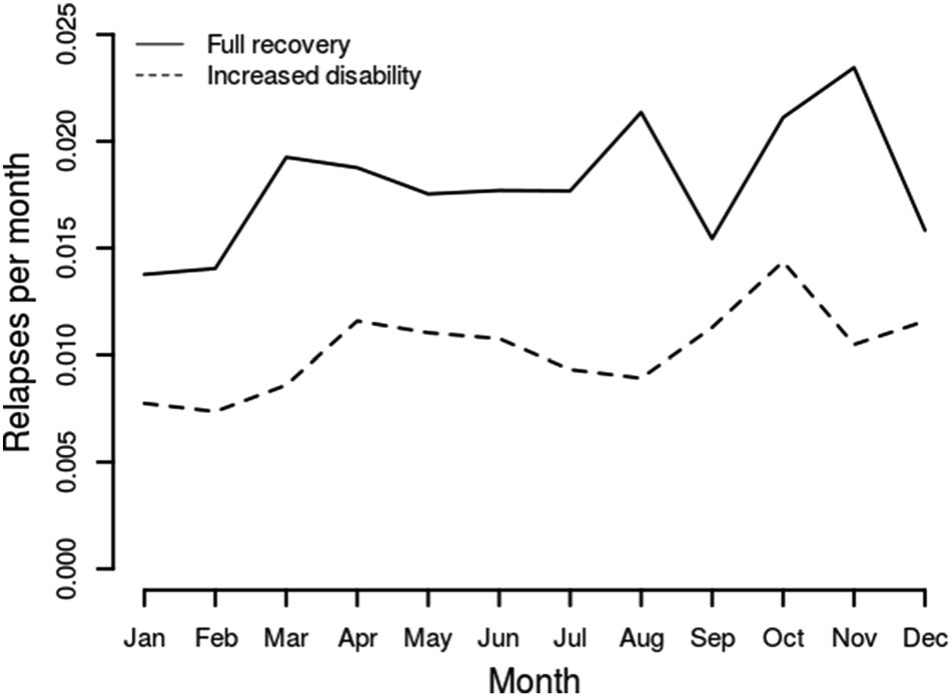
*Line graph* showing relapses per month observed, by recovery from the relapse

2729 relapses (70% of the total) were included in this analysis. Full recovery to the previous level of disability was observed in 1744, and 985 were followed by an increased level of disability compared to before relapse. Seasonal pattern was similar for both groups of relapse (Fig. 3). There was no association of recovery from a relapse with any climate variable (data not shown).

### Season of onset and time to EDSS milestones

There was a mean of 37 onset events in each month. Median time to EDSS 3.0 was 3.9 years, and to EDSS 4.0 was 5.7 years. There was no difference in time to EDSS 3.0 or 4.0 by season of onset. This remained the case after adjustment for age at onset, sex, and disease course in a Cox proportional hazards regression model (Table 4).

**Table 4:** Season of onset and EDSS milestones

	EDSS 3.0		EDSS 4.0	
*Median time to milestone*				
Onset in autumn	3.0 (2.1–4.1)		4.5 (3.3–7.8)	
Onset in spring	3.9 (3.0–5.7)		5.7 (4.9–7.6)	
Onset in summer	3.6 (2.5–4.8)		5.7 (4.2–7.5)	
Onset in winter	3.9 (3.2–4.8)		6.2 (4.8–7.6)	

Variable	Hazard ratio	*p*	Hazard ratio	*p*
*Cox proportional hazard regression*				
Onset in autumn	Reference		Reference	
Onset in spring	0.9 (0.6–1.2)	0.37	1.0 (0.7–1.4)	0.55
Onset in summer	0.9 (0.6–1.2)		0.9 (0.6–1.3)	
Onset in winter	0.9 (0.7–1.3)		0.9 (0.6–1.3)	
Age at onset	1.01 (1.00–1.03)	0.016	1.01 (0.99–1.03)	0.07
Male	1.3 (0.99–1.7)	0.06	1.3 (0.9–1.7)	0.14
Primary progressive	1.9 (1.3–2.8)	0.0007	3.0 (2.0–4.5)	<0.0001

## Discussion

In this northern European population-based study spanning a decade, we have shown a seasonal pattern in MS events, with a peak in late spring/early summer, and a trough in late summer. This pattern is most apparent in younger patients with relapsing–remitting disease and is associated with monthly hours of sunshine. However, this does not account for all variation observed, suggesting that seasonal patterns of MS events are multifactorial.

A seasonal pattern in MS relapses has previously been noted, with most studies reporting a solitary peak in spring and/or summer months in both northern [2, 17, 23, 26, 31] and southern hemispheres [7, 26], a pattern which has also been borne out by meta-analysis [11]. Fewer studies have detected a nadir in relapse rates, but in studies that have been able to detect this, the nadir tends to occur in late summer or autumn [9, 23, 26, 29, 31]. Three studies have not detected any seasonal variation [8, 18, 22]; however, one of these studies was a retrospective review of MS hospital admissions and thus may have overlooked less severe relapses [18], and the other two were relatively small studies in southern Europe where seasonal variation in climate may be less than in northern Europe or America [8, 22]. Indeed, a recent large study incorporating international data from the MS Base Registry suggests an effect of latitude on seasonal pattern of MS events [26]. These findings are also supported by our study, which is the largest single centre study of its kind and uses high-quality clinical data collected prospectively in a well-described cohort, suggesting that a seasonal pattern is genuine and not due to reporting bias.

There is also objective paraclinical evidence to support seasonal variation in neuroinflammatory activity; in a prospective North American study of 44 patients with parallel sequential MR imaging, new T2 activity was 2–3 times more frequent between March and August, although no similar increase in clinical events was observed [16]. Alterations in the pattern of seasonal cytokine variation have also been observed in MS patients: seasonal variation is seen in healthy controls in IL-4, IL-10, TNF-α, and IFN-γ, but in untreated MS patients, only seasonal variation in IL-10 was preserved, while in treated MS patients, the seasonal variation was lost for all four cytokines [27].

Since our study was based on prospectively collected data from individual patients, we have been able to assess risk of relapse at an individual level. This is a novel approach, which has allowed a more detailed analysis of monthly risk of an event than has previously been possible and has also allowed us to take into account the contribution of clinical variables to the probability of a relapse.

An association between UV levels and pattern of MS events has previously been observed [26, 29], and it has been proposed that this association occurs as a result of the mechanism of vitamin D production [29]. The best-fitting model in our study included hours of sunshine, although the mechanism by which this might take effect is not clear. We observed highest odds of relapse during early summer when hours of sunshine were high, and lowest odds of relapses during late summer. This implies that if vitamin D has a role, it cannot be the only factor that is relevant in triggering a relapse and may be modified by alternative environmental factors which modulate its effects.

Other factors associated with increased probability of relapse include younger age [30], shorter disease duration [30], and viral infections [1, 6], while pregnancy and breastfeeding [5, 14], serum vitamin D levels [25], and DMTs [3, 12, 19, 21] appear protective. It seems most likely that interaction between a number of these and other factors leads to initiation of CNS inflammation, manifesting clinically as a relapse. However, the relative contribution of individual factors and their contribution to the pathological sequence of events remain elusive. In addition, while some factors associated with probability of an MS event exhibit a seasonal pattern, such as climate variables or infections, others have a very different risk profile, such as the declining risk of relapse with increasing age [30], or the reduction in risk during pregnancy with a return to the previous levels of risk post-natally [5]. Therefore, it would seem unlikely that MS events would conform to a single pattern such as seasonal variation.

This study has a number of strengths. It is a large, population-based study, with good relapse ascertainment in a patient cohort that has had detailed clinical follow-up for more than 10 years. By confining the study to one geographical area, possible confounding effects of other environmental factors have been minimised. The analysis utilised objectively measured climate variables, and allowed clustering of multiple relapses within a given individual to be taken into account, thus allowing for individual variation in relapse rate.

The relapse rate in this study is considerably lower than that observed in most randomised controlled trials, which tend to select patients with active or highly active relapsing disease. This difference in relapse rate may also occur as the result of a more rigorous surveillance system. However, our data are comparable with rates seen in prospective population-based studies and we feel likely to be a true representation of overall relapse frequency in this cohort. We also recognise additional limitation in the analysis of these data; both seasonal variation and age-specific differences in relapse frequency may have occurred as a result of a systematic reporting bias. Although this is difficult to confidently exclude, it would seem unlikely given the rigorous methodology and results of previous studies.

In addition, relapses in this study have been grouped by month, and some excluded by necessity, because no accurate date could be ascribed. Patients with relapse of uncertain date appear to be more likely to have primary progressive disease, have shorter duration of follow-up, be older, and be male. However, this missing data should only bias conclusions if the relapses of uncertain date had a different seasonal pattern than those with a confirmed date. Only a limited number of climate variables were included here, and there may be other factors (or combinations of factors) which could explain remaining monthly variation in relapse rates. Our model also only adjusts for monthly (rather than daily) weather, and thus, some residual confounding effects could remain. It also adjusts for weather at the area level (with data on sunshine, rainfall, and temperature from south Wales, but UV index data for the UK as a whole) so may not represent geographical location on an individual level. Furthermore, there is no information on the amount of time each individual spent outside in a given month, and thus, individual exposure to climate variables is proxied by the monthly values of these variables, leading to measurement error in individual exposure.

Finally, the geographical limitations of this study, whilst minimising confounding by other, unmeasured environmental factors also minimise variation in exposure in this population. Stronger associations with climate variables might be observed in a population with a more diverse range of exposure, both by geography and across the year. All the climate variables except rainfall were very highly correlated, and thus, the independent associations of relapse rate with all climate variables could not be examined.

In conclusion, we have interrogated patterns of relapse in a large population-based cohort of patients from south east Wales with detailed clinical data, confirming the presence of a seasonal pattern in MS events and demonstrating association with hours of sunshine. However, in exploring the relative contribution of this and other factors to observed seasonal variation, we have recognised that the causes of a relapse are likely to be multifactorial and that a better understanding of the effect of environmental factors on the development of MS and its clinical manifestations remains to be established. This, in turn, may offer opportunities for improved treatments, strategies for relapse prevention, and alteration of risk of disease on a population level.
